# Topical Calcineurin Inhibitors: Expanding Indications for Corneal and Ocular Surface Inflammation

**DOI:** 10.18502/jovr.v14i4.5435

**Published:** 2019-10-24

**Authors:** Hamed Ghasemi, Ali Djalilian

**Affiliations:** ^1^ Eye Research Center, Farabi Eye Hospital, Tehran University of Medical Sciences, Tehran, Iran; ^2^ University of Illinois at Chicago, USA

The immune system protects the host against environmental and pathogenic insults while maintaining tolerance to self-antigens and commensal microbial flora. Inflammation and hyperactivity of the immune system play a central role in the pathophysiology of many ocular diseases such as vernal keratoconjunctivitis (VKC), dry eye disease, stromal keratitis, etc. Inflammation is the key target in the treatment of most cornea and ocular surface diseases.

Despite many advances in the last several decades, corticosteroids still remain the mainstay of therapy for anterior segment inflammation and are the most widely used topical anti-inflammatory drugs. However, unwanted ocular side effects such as glaucoma and cataract often preclude their use on a chronic basis. Calcineurin inhibitors such as cyclosporine A and tacrolimus are now commonly used as “steroid sparing” topical agents to prevent and treat diseases with T-cell-mediated pathophysiology. They specifically inhibit calcineurin which plays a central role in T-cell activation. Successful treatment of multiple refractory anterior segment inflammatory diseases has been reported with these agents. These conditions include prophylaxis and treatment of corneal graft rejection, chronic allergic keratoconjunctivitis, and ocular graft-versus-host disease.^[1,2,3,4,5]^

Both cyclosporin and tacrolimus are insoluble in water which creates a challenge for making a topical eye drop. Earlier studies often reported dissolving them in oil-based vehicles which were poorly tolerated by the patients. Emulsions and liposomal preparations appear to be better tolerated. As far as the choice between tacrolimus and cyclosporin A, we tend to favor tacrolimus given that it is more hydrophilic with a higher transcorneal diffusion rate than cyclosporine. The potency of tacrolimus is also 10–100 times higher than cyclosporine. These characteristics make tacrolimus theoretically more effective for the treatment of deeper corneal inflammation. While topical cyclosporin is available as a commercial preparation, a commercial preparation of topical tacrolimus eye drop is only available in a few countries (Japan, Brazil, etc.), and therefore it often needs to be compounded. We recommend a concentration of 0.03 to 0.05% for compounded tacrolimus. As most practitioner know, there is also a skin ointment preparation of tacrolimus (0.03% and 0.1%) that has been used in the eye. In patients who are unable to get compounded medication, we have prescribed the skin ointment off label and have them apply it to the lid margin. Finally, it is worth noting that there is a theoretical risk that topical application of tacrolimus could increase the risk of developing local cancers such as lymphoma, however, this has not been found to be the case on the eye.

In this issue of the *Journal of Ophthalmic and Vision Research*, Akbari et al have reported that addition of 0.05% topical tacrolimus to conventional treatment enhances visual acuity and reduces corneal inflammation, neovascularization, and scarring in eyes with herpetic stromal keratitis.^[6]^ This study highlights the utility of tacrolimus for deeper corneal inflammation. Likewise, the study by Chatterjee et al concluded that topical cyclosporine A 0.05% is effective and safe in Indian children with moderate to severe VKC with good steroid-sparing effect.^[7]^ This confirms previous observations that topical calcineurin inhibitors are highly effective for chronic allergic diseases which is well-known to be medicated by type 2 T helper (Th2) cells.

As mentioned earlier, one of the main limitations of both topical cyclosporin and topical tacrolimus is their tolerability which appears to be concentration dependent. Patients with significant ocular surface disease and dry eye appear to have the most trouble tolerating these medications. Strategies to improve the tolerance of calcineurin inhibitors include refrigerating the drops and using short-term topical steroids to control the inflammation prior to starting therapy. Future formulations are expected to improve the tolerance and bioavailability of these medications; thus, they will likely remain one of the important steroid-sparing agents for patients with inflammatory corneal and ocular surface diseases.
